# Absence of near-ambient superconductivity in LuH_2±*x*_N_*y*_

**DOI:** 10.1038/s41586-023-06162-w

**Published:** 2023-05-11

**Authors:** Xue Ming, Ying-Jie Zhang, Xiyu Zhu, Qing Li, Chengping He, Yuecong Liu, Tianheng Huang, Gan Liu, Bo Zheng, Huan Yang, Jian Sun, Xiaoxiang Xi, Hai-Hu Wen

**Affiliations:** National Laboratory of Solid State Microstructures, Department of Physics, Collaborative Innovation Center of Advanced Microstructures, Nanjing University, Nanjing, China

**Keywords:** Superconducting properties and materials, Superconducting properties and materials

## Abstract

A recent study demonstrated near-ambient superconductivity in nitrogen-doped lutetium hydride^1^. This stimulated a worldwide interest in exploring room-temperature superconductivity at low pressures. Here, by using a high-pressure and high-temperature synthesis technique, we have obtained nitrogen-doped lutetium hydride (LuH_2±*x*_N_*y*_), which has a dark-blue colour and a structure with the space group $Fm\bar{3}m$ as evidenced by X-ray diffraction. This structure is the same as that reported in ref. ^1^, with a slight difference in lattice constant. Raman spectroscopy of our samples also showed patterns similar to those observed in ref. ^1^. Energy-dispersive X-ray spectroscopy confirmed the presence of nitrogen in the samples. We observed a metallic behaviour from 350 K to 2 K at ambient pressure. On applying pressures from 2.1 GPa to 41 GPa, we observed a gradual colour change from dark blue to violet to pink-red. By measuring the resistance at pressures ranging from 0.4 GPa to 40.1 GPa, we observed a progressively improved metallic behaviour; however, superconductivity was not observed above 2 K. Temperature dependence of magnetization at high pressure shows a very weak positive signal between 100 K and 320 K, and the magnetization increases with an increase in magnetic field at 100 K. All of these are not expected for superconductivity above 100 K. Thus, we conclude the absence of near-ambient superconductivity in this nitrogen-doped lutetium hydride at pressures below 40.1 GPa.

## Abstract (web summary)

Nitrogen-doped lutetium hydrides LuH_2±*x*_N_*y*_ synthesized using a high-pressure and high-temperature synthesis technique did not show near-ambient superconductivity at pressures below 40.1 GPa.

## Main

Metallic hydrogen and hydrogen-rich materials provide interesting platforms for studying room-temperature superconductivity from the time it was proposed theoretically^2^. However, it is difficult to experimentally achieve high-temperature superconductivity (HTS) at low pressures^3,4^. Theorists proposed that polyhydrides may have the potential to realize HTS because of the effect of internal chemical pressure^5^. HTS was experimentally observed in H_3_S with a transition temperature (*T*_c_) above 200 K at high pressure (about 200 GPa) as theoretically predicted^6–8^. After that, other hydrogen-rich superconductors, such as LaH_10_ and CaH_6_, were discovered^9–15^. However, according to the Bardeen–Cooper–Schrieffer theory, HTS would rely on very strong electron–phonon coupling with a very high Debye temperature. According to McMillan’s formula, if we assume a Debye temperature of 500 K and a Coulomb screening constant μ^*^ = 0.13, the requested electron–phonon coupling constant *λ* would be as large as 12.2 for *T*_c_ = 100 K. This huge *λ* cannot provide a stable lattice structure. Thus HTS can be achieved in only systems that are protected by extremely high pressures.

Recently, superconductivity was reported at about 294 K in nitrogen-doped lutetium hydride at a pressure of about 1 GPa (ref. ^1^), which is interesting and important if the observation could be repeated. As reported in ref. ^1^, the dark-bluish ternary compound (with the formula LuH_3-*δ*_N_*ε*_) can be tuned to a near-ambient superconductor at a relatively low pressure (1–2 GPa), accompanied by a colour change from blue to pink and red. In previous experiments, superconductivity with much lower *T*_c_ was reported in lutetium hydrides at high pressures^16,17^. Thus, compared with the above results of lutetium hydrides, the discovery of near-ambient superconductivity in nitrogen-doped lutetium hydride is notable. It is interesting to study whether room-temperature superconductivity really exists in this nitrogen-doped lutetium hydride at relatively low pressures.

## Physical properties at ambient pressure

**Fig. 1:**
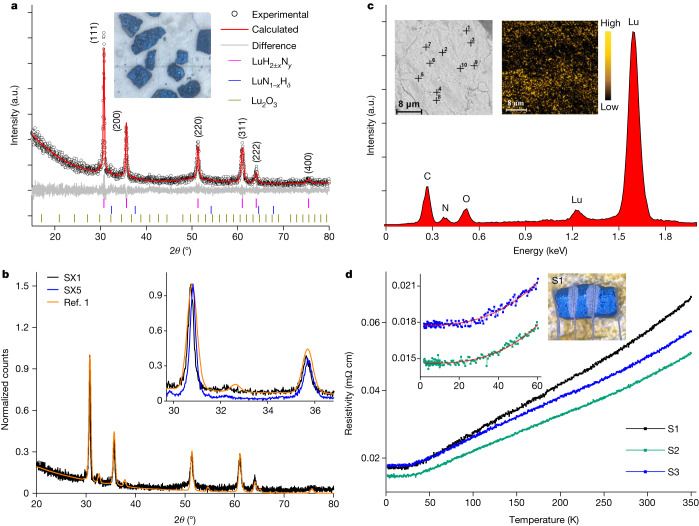
Structure, composition and transport measurements of LuH_2±*x*_N_*y*_. **a**, Powder XRD patterns of LuH_2±*x*_N_*y*_ and Rietveld fitting curves (red lines) to the data. The tiny reflection at 32.2° can be indexed to the phase of Lu_2_O_3_. Inset, LuH_2±*x*_N_*y*_ samples that exhibit a dark-blue colour. **b**, Comparison of XRD patterns with normalization of our sample SX1 to the data downloaded from ref. ^1^. Inset, enlarged view with 2*θ* from 29° to 37° for two of our samples (SX1 and SX5) and that from ref. ^1^. The main reflections of our samples seem to be sharper than those of ref. ^1^. **c**, SEM images and typical EDS of spot 1. Left inset, the image with 10 spots measured by point-wise measurement of EDS; right inset, the mapping image for nitrogen elements in another region. It is clear that the nitrogen distribution is not uniform in the sample, and it remains to be resolved at which positions these nitrogen atoms are located in the lattice. **d**, Temperature dependence of resistivity for three LuH_2±*x*_N_*y*_ samples under ambient pressure. Inset, the image of the measured sample (S1) with electrodes attached at ambient pressure. The error bar for determining resistivity is about ±10%. The *ρ–T* curves are roughly linear from 60 K to 300 K and exhibit a power-law temperature dependence at lower temperatures (2–60 K). The red solid lines represent the fitting curves using *ρ* = *ρ*_0_ + *AT*^*n*^ from 2 K to 60 K, where *ρ*_0_ is the residual resistivity and *n* and *A* are the fitting parameters. The fitting yields *n* = 2.89 and 2.71 for S2 and S3, respectively. a.u., arbitrary unit. Source data

**Extended Data Fig. 1:**
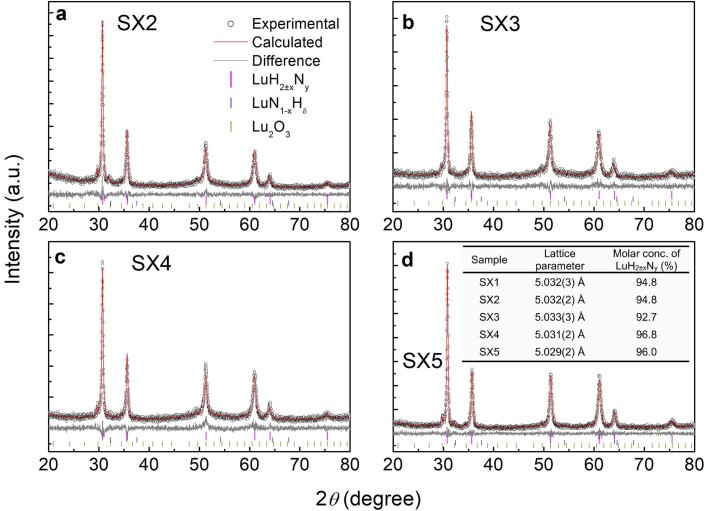
XRD and Rietveld refinement on other four samples. **a**–**d**, Powder X-ray diffraction patterns of four samples (SX2-SX5) and Rietveld fitting curves (red lines) to the data. The inset in (**d**) presents the Rietveld fitting results (lattice parameters and molar concentration of LuH_2±x_N_y_) of five samples (SX1-SX5). The slight difference of the lattice constants may be attributed to the different hydrogen/nitrogen concentrations or the crystallinity in different samples. The Rietveld fitting to these results shows a high purity with the main phase in a molar ratio of about 95%, and are highly consistent with each other. Source data

Figure 1a shows the X-ray diffraction (XRD) patterns and Rietveld refinement of the LuH_2±*x*_N_*y*_ sample. As shown in Fig. 1a, the experimental data can be well fitted to the structure of LuH_2_ with the space group ${Fm}\bar{3}m$ and lattice parameter *a* = 5.032(3) Å (the value in parentheses represents the uncertainty of the last digit). LuH_3_ with a face-centred cubic structure is not stable at ambient pressure, but a stable hexagonal structure with a space group $P\bar{3}c1$ was reported previously^18,19^. As shown in Fig. 1b, the main reflections of XRD data in our sample SX1 and those downloaded from ref. ^1^ almost coincide, indicating that they have a similar structure. The lattice constant of our sample SX1 is about *a* = 5.032(3) Å, which is slightly larger than the *a* = 5.0289 Å reported in ref. ^1^, but it is very close to the value of 5.033 Å determined previously^19,20^ for LuH_2_. For checking the lattice constants of our samples more carefully, we did a series of XRD measurements on different samples and found that the lattice constant ranges from 5.029(2) Å to 5.033(3) Å (Extended Data Fig. 1). Compared with the samples in ref. ^1^, our samples show fewer impurities. Therefore, according to our XRD data, we obtained compounds that have almost the same structure as that reported in ref. ^1^.

**Extended Data Fig. 2:**
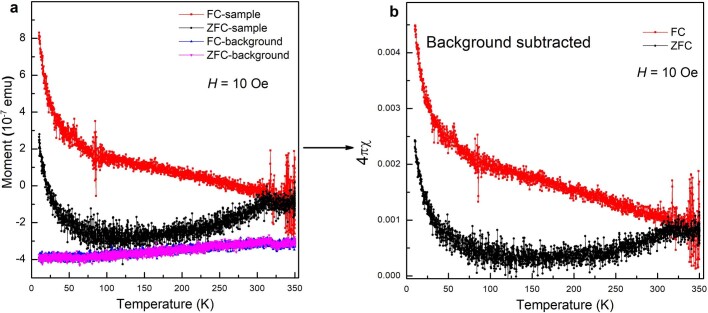
Temperature dependence of the magnetic susceptibility for LuH_2±x_N_y_ at ambient pressure. **a**, Temperature dependence of magnetic moment for LuH_2±x_N_y_ (upper part) along with background signal (lower part) measured in an applied field of 10 Oe using both the ZFC and FC modes. **b**, The corresponding magnetic susceptibility 4πχ with the background subtracted, for a superconductor we should expect 4πχ = −1. In calculating the magnetic susceptibility, we have used a density of 9.22 g/cm^2^ quoted for compound LuH_2_. The measurements were carried out by using the SQUID-VSM with a vibrating amplitude of 5 mm and frequency of 13.01 Hz. Source data

Energy-dispersive X-ray spectroscopy (EDS) is used to analyse the elemental composition in the sample. Figure 1c (left inset) shows a scanning electron microscope (SEM) image of LuH_2±*x*_N_*y*_ with 10 randomly measured spots marked by black crosses. The nitrogen composition at these spots is given in Extended Data Table 1. The typical EDS of spot 1 is shown in Fig. 1c, in which a weak peak from nitrogen can be identified. Figure 1c (right inset) shows the spatial distribution of nitrogen. The nitrogen distribution seems to be widespread in the entire area and locally inhomogeneous. Because it is impossible to detect the hydrogen atoms by EDS, and XRD shows that the structure is consistent with LuH_2_, we define the chemical formula of our samples as LuH_2±*x*_N_*y*_. Figure 1d shows the temperature dependence of resistivity (*ρ–T*) for three samples of LuH_2±*x*_N_*y*_ at ambient pressure. All samples show metallic behaviour down to 2 K. Magnetization was measured for the samples at 10 oersted (Oe) in the zero-field-cooling (ZFC) and field-cooling modes. The signal is positive and usually very small (Extended Data Fig. 2).

## Raman spectroscopy

**Fig. 2:**
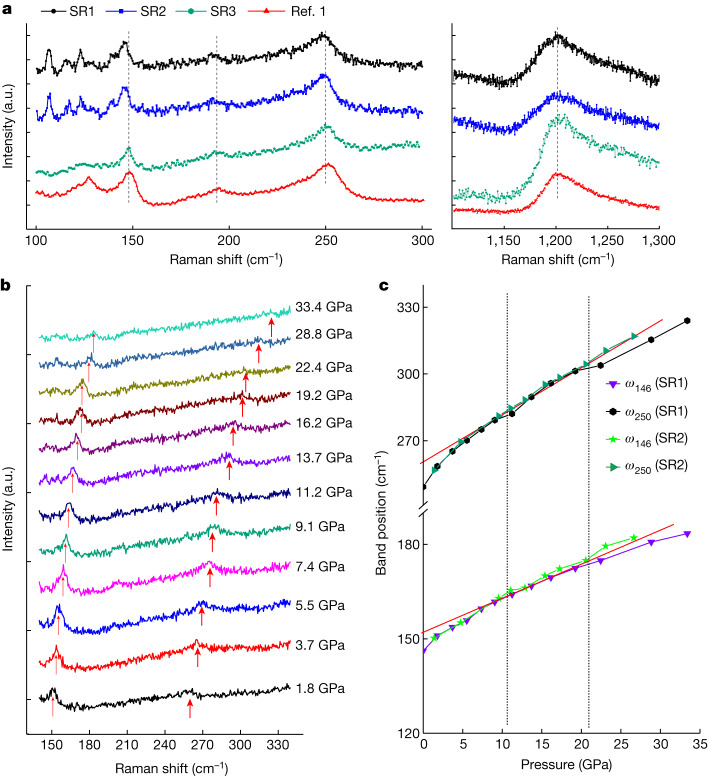
Raman spectra of LuH_2±*x*_N_*y*_. **a**, Typical Raman spectra collected at ambient pressure for three samples (black, blue and green curves), and the data of nitrogen-doped lutetium hydride from ref. ^1^ (red curve) are shown for comparison. The samples are labelled SR1, SR2 and SR3, respectively. The tiny peaks of SR1 and SR2 below 140 cm^−1^ originate from the background signal of Raman spectrometer 1 (Extended Data Fig. 3). The band positions in the spectrum below 300 cm^−1^ have slightly smaller wavenumbers compared with those in ref. ^1^, indicating that the lattice constant of our sample is slightly larger. The results are also consistent with the lattice parameters obtained from XRD. This slight variation may be attributed to the different hydrogen and nitrogen concentrations in different samples. **b**, Raman spectra were collected at high pressures using Raman spectrometer 1 with a mixture of methanol, ethanol and water as the pressure medium for SR1. **c**, Evolution of band positions of Raman spectra under different pressures. The two curves correspond to the bands as indicated in **b** by red arrows. The red lines are guides for the eyes to show the change in the slopes. a.u., arbitrary unit. Source data

**Extended Data Fig. 3:**
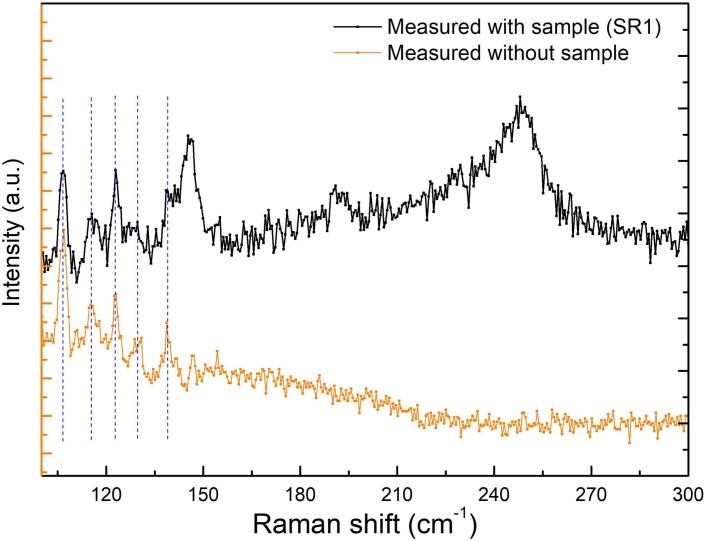
Raman spectra collected on Raman spectrometer #1. The black curve (upper one) shows the raw data measured on the LuH_2±x_N_y_ sample (SR1) using the Raman spectrometer #1, while the orange curve (bottom one) shows the background signal of the instrument without the sample nor the DAC. It is clear that the bands around 106 cm^−1^, 115 cm^−1^, 122 cm^−1^, 129 cm^−1^ and 138 cm^−1^ are originated from the instrument itself. Source data

We collected Raman spectra of our LuH_2±*x*_N_*y*_ samples at ambient pressure using two different instruments—named Raman spectrometers 1 and 2—both with 532-nm laser excitations (Methods). Raman spectra measured for three of our samples at ambient pressure are shown in Fig. 2a; the data from ref. ^1^ are shown for comparison (red curve). Raman spectra of our samples (SR1–SR3) almost coincide with each other, indicating uniform crystallinity. Moreover, the band positions of Raman spectra at around 150 cm^−1^, 190 cm^−1^, 250 cm^−1^ and 1,200 cm^−1^ (Fig. 2a, dashed lines) in our samples are highly consistent with those reported in ref. ^1^, which indicates that our samples are similar to those in ref. ^1^. We also notice that the spectra collected using Raman spectrometer 1 on samples SR1 and SR2 below 140 cm^−1^ deviate from those reported in ref. ^1^. In ref. ^1^, only one peak was observed in the range 100–140 cm^−1^, but in our samples SR1 and SR2, there are several tiny peaks. After checking Raman spectrometer 1 carefully, we find that these tiny peaks below 140 cm^−1^ are extrinsic and are because of the instrument (Extended Data Fig. 3). To prove this further, we used Raman spectrometer 2 to measure another sample (SR3), which is shown as a green curve in Fig. 2a. We can see that the Raman spectrum of sample SR3 is similar to that reported in ref. ^1^. However, Raman spectrometer 2 is not suitable for measurements involving high pressure because of a limitation of its objective lens at short working distances.

**Extended Data Fig. 4:**
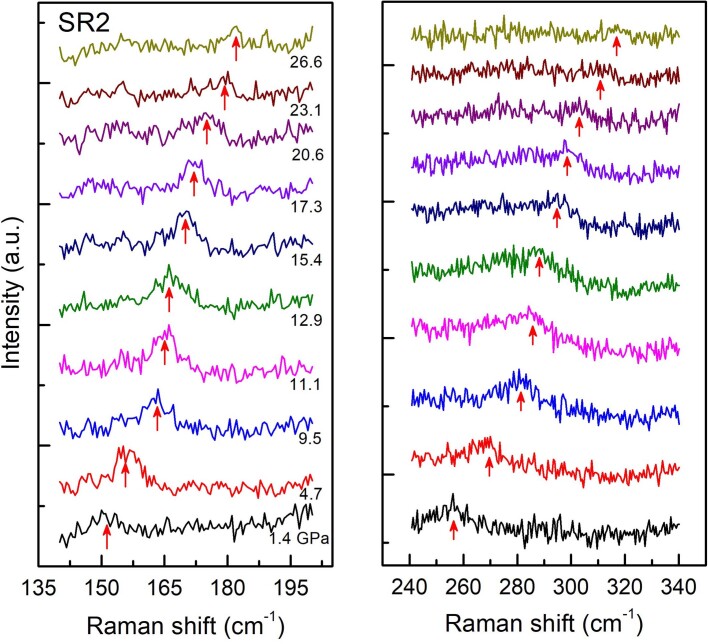
Raman spectra of another LuH_2±x_N_y_ sample (SR2) under different pressures. In this measurement, a rhenium gasket was used. The Raman bands around 146 cm^−1^ and 250 cm^−1^ show continuous increase with increasing pressure. Due to a sizeable Raman signal from the instrument under low wavenumbers, we show only the data above 140 cm^−1^. Source data

Figure 2b shows the Raman spectrum of the LuH_2±*x*_N_*y*_ sample (SR1) under various pressures up to 33.4 GPa. Because the Raman band of 1,200 cm^−1^ overlaps with the characteristic band of diamond anvils at high pressures, and the one at around 190 cm^−1^ is rather weak, we focus on only the two bands at around 146 cm^−1^ and 250 cm^−1^. On compression, these two Raman bands shift to higher wavenumbers, suggesting a sizeable change of interatomic interaction with pressure. We also conducted an independent control experiment up to 26.6 GPa for another LuH_2±*x*_N_*y*_ sample (SR2) (Extended Data Fig. 4). Both sets of data show good agreement with each other.

We then extracted the wavenumbers of these two bands in the Raman spectra under different pressures and summarized the pressure-dependent band positions in Fig. 2c. The frequencies of Raman bands at around 146 cm^−1^ and 250 cm^−1^ show a continuous increase with increasing pressure. With careful analysis of the data, we found that the slope of the two curves changes markedly around 10 GPa, as shown by the deviation from the red solid lines in Fig. 2c. There is another slight change at around 20 GPa. In ref. ^1^, the abnormal changes of Raman band shift under pressure were correlated with three distinct phases. The anomalous Raman band shifts of our samples at high pressures seem to be consistent with those reported in ref. ^1^, except for the different thresholds of pressure at which the changes in slope occur. These changes in slope are not necessarily associated with three distinct phases, but all phonons are unharmonic, and they tend to react to compression more steeply at low pressure and less steeply at high pressure.

## Resistance and colours at high pressures

**Fig. 3:**
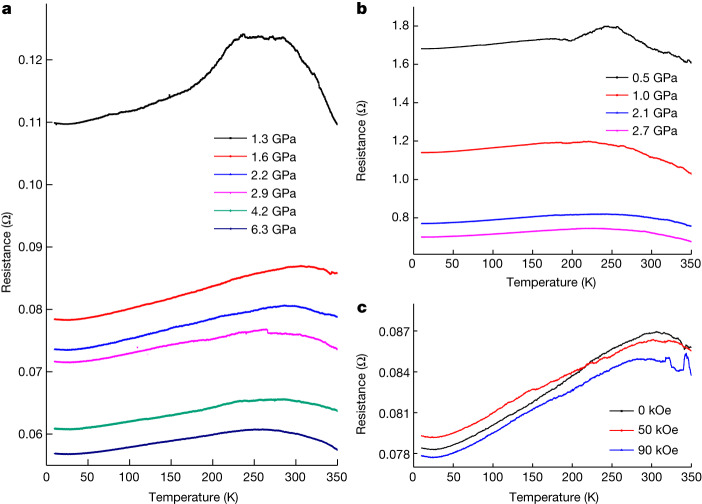
Temperature-dependent resistance of LuH_2±*x*_N_*y*_ at different pressures up to 6.3 GPa. **a**, Temperature dependence of the electrical resistance of LuH_2±*x*_N_*y*_ from 10 K to 350 K with pressures up to 6.3 GPa for run 1 (diamond-anvil cell (DAC) filled with polycrystalline pieces). The weak upturn of the *R*(*T*) curve in the low-temperature region may be because of the hopping of electrons through a large inter-grain spacing or grain boundaries when the grains are compacted loosely in the DAC space. This explanation is supported by the weakening and absence of this low-temperature upturn when the pressure increases. **b**, Temperature dependence of the electrical resistance of LuH_2±*x*_N_*y*_ up to 2.7 GPa for run 2 with the DAC filled with the powder of the sample. Now the low-temperature upturn disappears. **c**, Temperature dependence of the electrical resistance of LuH_2±*x*_N_*y*_ measured at different magnetic fields up to 90 kOe at 1.6 GPa for run 1 (DAC filled with polycrystalline pieces). Source data

Figure 3a,b shows the temperature dependence of resistance from 10 K to 350 K under different pressures. The resistance at room temperature progressively decreases with an increase in pressure up to 6.3 GPa. The temperature-dependent resistance *R*(*T*) curve shows a universal hump structure around 300 K, which becomes weaker at higher pressures. This hump reflects a metal-to-semiconductor transition, which may share the same origin as that observed in hydrides of many other rare-earth elements^21,22^. To check whether the decrease in resistance around room temperature is related to a possible superconducting transition, we also measured *R*(*T*) curves of LuH_2±*x*_N_*y*_ at various magnetic fields at a pressure of 1.6 GPa. As shown in Fig. 3c, the resistance at varying magnetic fields exhibits a non-systematic evolution and does not show any drifting to lower temperatures as expected for a superconductor in a magnetic field.

**Fig. 4:**
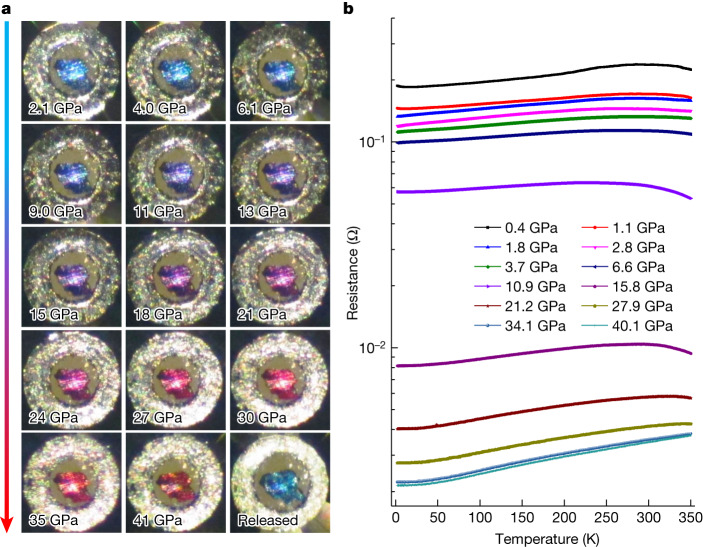
Pressure-induced colour change and evolution of temperature-dependent resistance of LuH_2±*x*_N_*y*_ at different pressures. **a**, The optical microscope images of LuH_2±*x*_N_*y*_ at different pressures up to 41 GPa. A colour change from dark blue to violet and pink-red is observed. **b**, Temperature dependence of the electrical resistance of LuH_2±*x*_N_*y*_ from 2 K to 350 K with pressures up to 40.1 GPa. In most *R*(*T*) curves, we can see a metallic behaviour from an intermediate temperature down to 2 K, either in the dark blue or the pink-red states. The *R*(*T*) curves at low pressures, such as at 0.4 GPa and 1.1 GPa, show a weak upturn in the low-temperature region, which gradually becomes invisible when the pressure is increased. There is a resistivity hump in the region around 300 K in the low-pressure region. Source data

**Extended Data Fig. 5:**
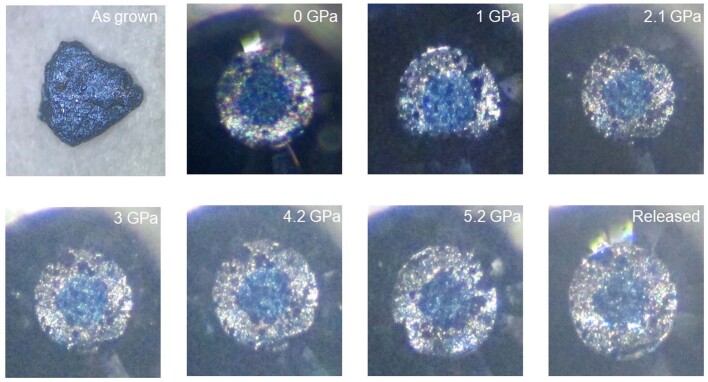
The optical microscope images of LuH_2±x_N_y_ at different pressures up to 5.2 GPa. In this run, the sample was directly filled in the DAC chamber without any pressure medium.

**Extended Data Fig. 6:**
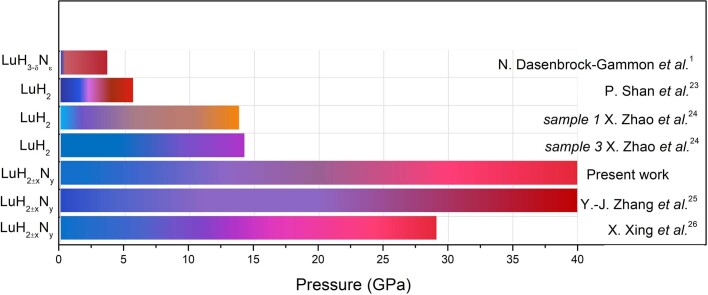
A sketch for pressure dependence of the color change for different samples. The data are collected from Fig. 4a and refs. ^1,23–26^.

One of the most notable phenomena reported in ref. ^1^ is the colour change from dark blue to pink and red with increasing pressure. The near-ambient superconductivity was suggested to occur in the state with a pink colour. We also tried to see this colour change in our samples with pressures up to 5.2 GPa (Extended Data Fig. 5), but the dark-blue colour was maintained. The pressure threshold for the colour change seems to be sample dependent in the nitrogen-free^23,24^ or nitrogen-doped samples^25,26^. Therefore we tried another run of optical measurements with pressures up to 41 GPa. In this case, the colour change could be clearly seen. As shown in Fig. 4a, the colour gradually changes from dark blue to violet, and then to pink-red. The crossover from dark blue to pink-red occurs in the region of about 11–21 GPa; after that the colour stays as pink-red. The correlation between the colour change and pressure for different samples is shown in Extended Data Fig. 6. We found that the pressure region of the colour change was consistent with the Raman band-shift anomaly shown in Fig. 2c. Although there is a recent study on photochromism of LnH_2+*x*_O_*y*_ (Ln = lanthanide element)^27^, which suggests that this effect may lead to the pink colour, we argue that this is not the reason for the pink-red colour in our samples because it gradually emerges with increase in pressure, and the pink-red colour appears almost in the entire sample at high pressure. This colour change may be explained by the shift of the plasma edge of a metal^24^ with a proper charge-carrier density; the latter can be easily tuned by pressure in systems containing shallow bands. Given the colour change from dark blue to pink-red, it is interesting to know whether the claimed superconductivity in ref. ^1^ can be found at this high-pressure range, especially in samples with a pink-red colour.

**Extended Data Fig. 7:**
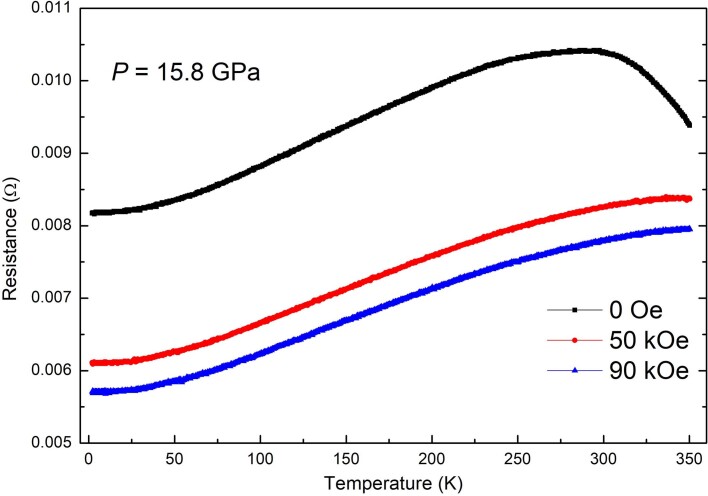
Temperature dependence of electrical resistance for LuH_2±x_N_y_. The measurements were conducted under a pressure of 15.8 GPa with various magnetic fields up to 90 kOe. The hump structure around 250–300 K is gradually suppressed by external fields and a negative magnetoresistance is observed. No superconducting signal was detected down to 2 K. Source data

We then carried out a new run of measurements on resistivity from 0.4 GPa to 40.1 GPa (Fig. 4b). The general behaviour is metallic for all the states at all pressures. We also measured the temperature-dependent resistance at 15.8 GPa for three different magnetic fields (0 kOe, 50 kOe and 90 kOe) and found a negative magnetoresistance, which contradicts the expectation for a superconducting state (Extended Data Fig. 7). Thus we can safely conclude that no superconductivity is observed at pressures below 40.1 GPa and above 2 K on the basis of resistance measurements.

## Magnetic moments at high pressures

**Fig. 5:**
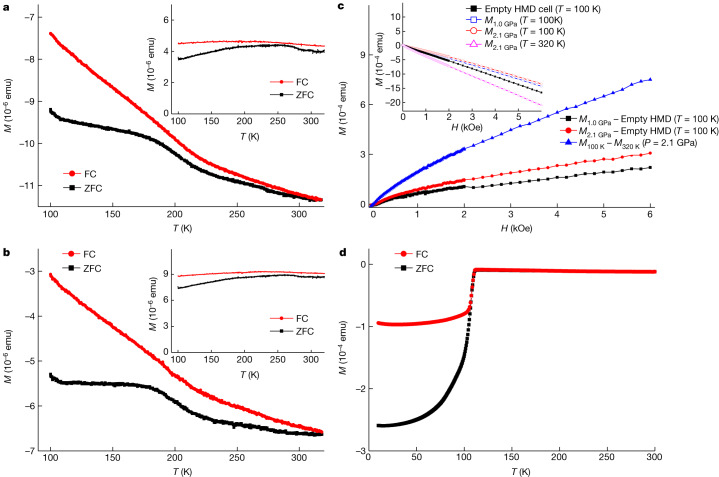
Magnetic properties for LuH_2±*x*_N_*y*_ at different pressures. **a**,**b**, Temperature dependence of magnetic moment (*M*) for LuH_2±*x*_N_*y*_ at pressures of 1 GPa (**a**) and 2.1 GPa (**b**). The raw data are shown in the main panels. Insets, corresponding magnetic moments measured in ZFC and field-cooling (FC) modes with the background subtracted. **c**, *M*(*H*) curves with different background signals subtracted. Inset, raw data of *M*(*H*) curves at 100 K at pressures of 1.0 GPa (open square) and 2.1 GPa (circle), and one curve at 320 K under 2.1 GPa (upward triangle). The *M*(*H*) curve measured at 100 K for the empty HMD cell is also shown (solid square). **d**, Temperature dependence of ZFC–FC magnetizations measured on a superconducting sample (Cu,C)Ba_2_Ca_3_Cu_4_O_11+*δ*_ (*T*_c_ = 112 K) with the same measured volume as that for LuH_2±*x*_N_*y*_ shown in **a** and **b**; the same HMD set-up was used under the same magnetic field (*H* = 60 Oe; *P* = 0.3 GPa). If the phase LuH_2±*x*_N_*y*_ were superconductive, one should observe a diamagnetic signal in the same scale at the same magnetic field (60 Oe). Source data

**Extended Data Fig. 8:**
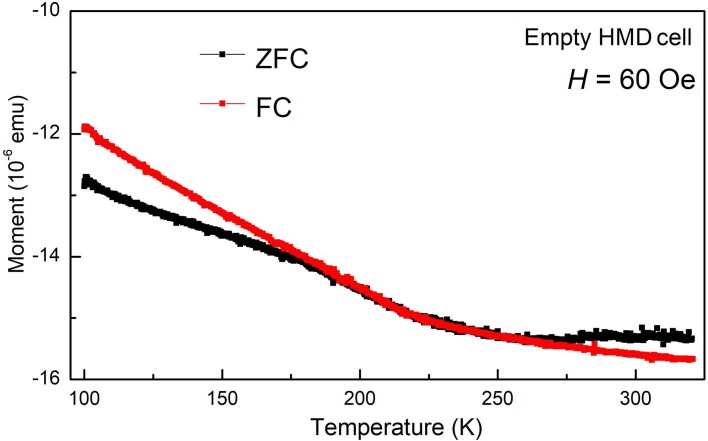
Temperature dependence of magnetic moment for the empty HMD cell. The applied magnetic field was *H* = 60 Oe. The *M*(T) curves with ZFC and FC modes are used as the background signals to obtain the magnetic moment purely from the sample in the DC magnetization measurements. Source data

**Extended Data Fig. 9:**
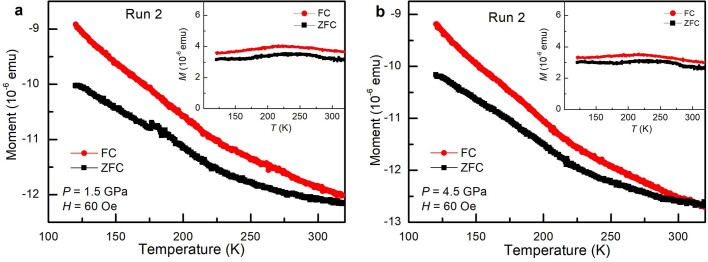
Temperature dependence of magnetic moment for LuH_2±x_N_y_. All curves were measured in an applied field of 60 Oe using both the ZFC and FC modes in run 2 at pressures of (**a**) 1.5 GPa and (**b**) 4.5 GPa. The raw data are shown in the main panels. The insets show the corresponding magnetization with the background signal removed. The magnetic susceptibility for LuH_2±x_N_y_ is positive in the whole temperature region, and no diamagnetic signal was detected, which is contrary to the existence of superconductivity. Source data

To check whether there is a diamagnetic signal because of the Meissner effect of the possible superconductivity in our as-grown samples, we measured the temperature-dependent d.c. magnetization *M*(*T*) curves of LuH_2±*x*_N_*y*_ at 1 GPa and 2.1 GPa. The sample volume for the high-pressure measurement is about 0.037 mm^3^. The *M*(*T*) curves at 60 Oe (the field used in ref. ^1^) at different pressures are shown in Fig. 5a,b. The magnetic moment increases with decreasing temperature and does not show a sudden drop in behaviour. Furthermore, owing to the background signal of the magnetization-measurement device Honest Machinery Designer (HMD), the values of the total magnetic moment are negative in the entire measured temperature region. To get the magnetization signal exclusively from the sample, we also measured the background signal of the HMD cell at 60 Oe. The data of the background signal are presented in Extended Data Fig. 8. The net magnetic moments after removing the related background are shown in Fig. 5a,b (insets). The net signal of magnetic moment is positive and very weak with a roughly flat feature in the 100–320 K temperature region. Figure 5c (inset) shows the isothermal magnetization *M*(*H*) curves for LuH_2±*x*_N_*y*_ at 100 K at 1 GPa (open squares) and 2.1 GPa (open circles). The *M*(*H*) curve shows a roughly linear behaviour with a negative slope from 0 kOe to 6 kOe, which is because of the background. To prove this point, we have measured one *M*(*H*) curve at 320 K at 2.1 GPa (upward triangle), and another at 100 K for the empty HMD cell (solid square). In Fig. 5c, we show the net *M*(*H*) curves after subtracting the related backgrounds. All net *M*(*H*) curves exhibit a roughly linear behaviour with a positive correlation. This corresponds to possible paramagnetic behaviour. We also conducted magnetization measurements on another LuH_2±*x*_N_*y*_ sample at pressures up to 4.5 GPa (Extended Data Fig. 9). The same HMD cell was successfully used to detect a clear superconducting transition in Bi samples previously^28^. To prove that this set-up is equally sensitive for detecting a superconducting phase in the high-temperature region, we carried out magnetization measurements on a superconducting sample (Cu,C)Ba_2_Ca_3_Cu_4_O_11+*δ*_ (*T*_c_ ≈ 112 K)^29,30^ (Fig. 5d). We can see that the signal is negative and huge compared with that of LuH_2±*x*_N_*y*_. Our magnetization measurements combined with the data of either temperature dependence or the isothermal magnetization curves show that there is no trace of near-ambient superconductivity in LuH_2±*x*_N_*y*_. The absence of near-ambient superconductivity in nitrogen-doped lutetium hydrides is supported by recent theoretical calculations^31–34^.

In summary, we have successfully synthesized nitrogen-doped lutetium hydrides LuH_2±*x*_N_*y*_ with a dark-blue colour. Although our synthesis method is different, the XRD and Raman spectroscopy confirmed that our samples have a structure similar to that reported in ref. ^1^, with a slight difference in lattice constant. Furthermore, the existence of nitrogen in our samples is also confirmed by EDS analysis. We also saw a colour change from dark blue to violet to pink-red on applying high pressures, although the threshold pressures for the colour change are higher than that used in ref. ^1^. Our resistivity measurements show the absence of superconductivity in LuH_2±*x*_N_*y*_ at pressures up to 40.1 GPa with all different colours down to 2 K. The magnetization measurements further prove that no superconductivity exists in LuH_2±*x*_N_*y*_ above 100 K under near-ambient pressures.

## Methods

### Sample preparation and characterization

We synthesized polycrystalline samples of LuH_2±*x*_N_*y*_ using a piston-cylinder-type high-pressure apparatus (LP 1000-540/50, Max Voggenreiter). NH_4_Cl and excessive CaH_2_ were used as the source for nitrogen and hydrogen, according to the chemical equation 2NH_4_Cl + CaH_2_ → CaCl_2_ + 2NH_3_ + H_2_. NH_4_Cl (Alfa Aesar 99.99%) was mixed well with CaH_2_ (Alfa Aesar 98%) in a molar ratio of 2:8 and pressed into a tablet. Then, the tablet made of Lu pieces (purity 99%, Grirem Advanced Materials) with a silver colour were separated from the NH_4_Cl + CaH_2_ tablet by a BN pellet and sealed into a gold capsule. The Lu pellet in each sintering weighs about 100 mg. Then the gold capsule was placed in a BN capsule and heated to 300–350 °C and held for 10 h at 2 GPa. Finally, we find that the Lu tablet turns into a new form composed of two well-separated different regions with dark-blue and silver colours, respectively; the dark-blue region corresponds to the LuH_2±*x*_N_*y*_ phase.

XRD measurements were performed on a Bruker D8 Advanced diffractometer with Cu*K*_*α*_ radiation. Rietveld refinements were done using TOPAS4.2 (ref. ^35^). The SEM photograph and the energy-dispersive X-ray microanalysis spectrum were obtained by Phenom ProX (Phenom) at an accelerating voltage of 15 kV. Unpolarized Raman-scattering experiments were performed using two instruments at room temperature, both with a 532-nm laser excitation line—one is a Raman spectroscopy system (LabRAM HR Evolution Horiba Jobin Yvon) and the other is a custom-built confocal microscopy set-up in the back-scattering geometry in which the scattered light was directed through Bragg notch filters. For clarity, we call the first Raman system Raman spectrometer 1 and the second Raman spectrometer 2. Before taking measurements, both systems were calibrated for wavenumbers by following the instrument instructions. To get valid data in the low-wavenumber region (below 140 cm^−1^), we used Raman spectrometer 2 for the Raman-scattering measurement on a new sample (SR3) placed in vacuum. Before measuring sample SR3, we checked Raman spectrometer 2 without a sample and found a smooth background without any band-like features in the low-wavenumber region. For measurements using Raman spectrometers 1 and 2, the laser power was 7.5 mW and 4 mW and the collection time was 60 s and 120 s, respectively, and all spectra were measured twice to check for reproducibility. For high-pressure Raman-spectra measurements, two runs of experiments were performed on Raman spectrometer 1 by using a DAC with T301 steel or rhenium as gasket and a mixture of methanol, ethanol and water as the pressure medium.

### Physical-property measurements at ambient and high pressures

Temperature-dependent resistivity and resistance measurements under ambient and high pressures were carried out with a physical-property measurement system (PPMS-9T, Quantum Design). The high pressure was generated by a DAC made of BeCu alloy with two opposing anvils. A four-probe van der Pauw method with platinum foil as electrodes was applied for resistance measurements. The d.c. magnetization measurements were performed with a SQUID-VSM-7T (Quantum Design). The d.c. magnetic moment measurements at high pressures were accomplished by using the DAC (attachment to a physical-property measurement system) designed by HMD. The sample is loaded in a hole in the middle of the gasket made of BeCu that needs pre-pressurization before high-pressure measurements. The anvils with bevelled culet sizes of 400 μm and 600 μm were used to generate high pressures. NaCl and Daphne 7373 were used as the pressure-transmitting medium during the resistive and magnetic-susceptibility measurements, respectively. For optical measurements, we used KBr as the pressure-transmitting medium. The pressure was measured at room temperature using the ruby fluorescence method^36^.

### Experimental reproducibility and controls

We have conducted various types of experiment on more than 30 samples: seven specimens were used for measuring resistivity at ambient pressure; five specimens for resistivity at high pressure; two specimens for magnetization measurements at ambient pressure; three specimens for magnetization measurements at high pressure; six specimens for XRD measurements; three runs (specimens) for the Raman spectroscopy measurements; five runs (specimens) for optical image measurements; and eight specimens for SEM and EDS measurements.

## Online content

Any methods, additional references, Nature Portfolio reporting summaries, source data, extended data, supplementary information, acknowledgements, peer review information; details of author contributions and competing interests; and statements of data and code availability are available at 10.1038/s41586-023-06162-w.

### Source data

Source Data Fig. 1

Source Data Fig. 2

Source Data Fig. 3

Source Data Fig. 4

Source Data Fig. 5

Source Data Extended Data Fig. 1

Source Data Extended Data Fig. 2

Source Data Extended Data Fig. 3

Source Data Extended Data Fig. 4

Source Data Extended Data Fig. 7

Source Data Extended Data Fig. 8

Source Data Extended Data Fig. 9

## Figures and Tables

**Extended Data Table 1:**
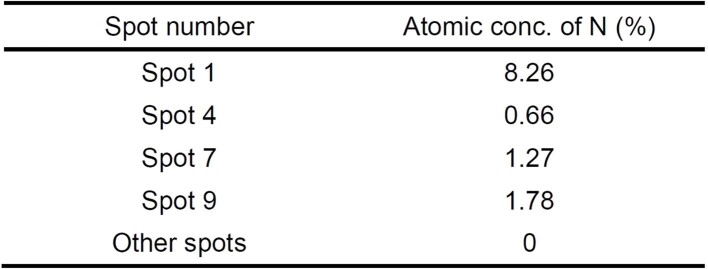
The atomic concentration of nitrogen by EDS analysis at 10 spots of the sample

The average value of nitrogen in atomic ratio over these 10 spots is about 1.2%.

## Data Availability

All data needed to evaluate the conclusions are available in the paper. Source data are provided with this paper.
